# Expansion of atypical memory B cells is a prominent feature of COVID-19

**DOI:** 10.1038/s41423-020-00542-2

**Published:** 2020-09-02

**Authors:** Barbara Oliviero, Stefania Varchetta, Dalila Mele, Stefania Mantovani, Antonella Cerino, Cesare G. Perotti, Serena Ludovisi, Mario U. Mondelli

**Affiliations:** 1 Division of Infectious Diseases and Immunology, Fondazione IRCCS Policlinico San Matteo, Pavia, Italy; 2 Immunohaematology and Transfusion Service, Fondazione IRCCS Policlinico San Matteo, Pavia, Italy; 3 Department of Internal Medicine and Therapeutics, University of Pavia, Pavia, Italy

**Keywords:** Immunology, Viral infection

Severe acute respiratory syndrome coronavirus 2 (SARS-CoV-2) is an RNA virus responsible for a pandemic that causes asymptomatic or paucisymptomatic infection in most individuals but may be responsible for severe interstitial pneumonia, myocarditis, acute kidney injury, hepatitis, acute respiratory distress syndrome (ARDS), multiorgan failure (MOF), and death.^1^ Patients with severe coronavirus disease (COVID-19) typically show signs of hyperinflammation characterized by an exuberant host immune response leading to a virus-driven cytokine release syndrome.^2^ A hallmark of COVID-19 is lymphopenia, involving virtually all cell lineages, and a number of studies have provided evidence in support of altered innate and adaptive immune responses in COVID-19.^2^ With respect to the latter, most studies focused on phenotypic and molecular T-cell alterations; however, little or no information is available for B cells, apart from the possible role of SARS-CoV-2-specific neutralizing antibodies in controlling the infection and clinical outcome. An altered distribution of B-cell subsets is usually observed in chronic viral infections, including hepatitis C and B^3,4^ and HIV.^5^ In an effort to identify characteristic changes that may be associated with favorable or poor outcomes in patients with COVID-19, we studied peripheral blood B-cell subsets in 17 patients presenting with severe SARS-CoV-2 infection and interstitial pneumonia with a viral RNA-positive nasopharyngeal swab or bronchoalveolar lavage. All were admitted to the hospital because of severe dyspnea requiring at least a Venturi type of mask for oxygen ventilation. Four patients required a ventilation upgrade to continuous positive airway pressure with high positive end-expiratory pressure or transfer to the ICU and intubation. Ten of these patients died because of ARDS and/or MOF. Follow-up peripheral blood mononuclear cells were available from seven recovered patients ~45 days after discharge. Seven convalescent (Conv) individuals who donated hyperimmune plasma because of serum high-titer neutralizing antibodies immediately after hospital discharge or after home quarantine were also investigated. Laboratory findings are reported in Supplementary Table 1. Thirteen clinically healthy, SARS-CoV-2 RNA-negative subjects of the same age group as the patients served as controls (HD). The study was approved by the Institutional Review Board of Fondazione IRCCS Policlinico San Matteo (prot. N. 20200038910).

B cells (CD19+) were examined by flow cytometry (FACS Celesta, BD Bioscience, San Diego, CA, USA) and are shown as frequencies and absolute numbers that largely overlapped, with only a few exceptions. B cells were categorized according to the expression of CD21, CD27, and CD10 molecules as immature transitional (itBC, CD27^−^/CD10^+^), atypical memory (atMBC, CD21^lo^/CD27^−^/CD10^−^), activated memory (AMBC, CD21^lo^/CD27^+^/CD10^−^), classical memory (cMBC, CD21^+^/CD27^+^/CD10^−^), and naïve (nBC, CD21^+^/CD27^−^/C10^−^) cells (Fig. 1a and Supplementary Fig. 1). Analysis of B-cell subsets revealed a characteristic and significant expansion of atMBC in terms of both frequencies and numbers, in patients with COVID-19 (CoV-2) compared with HD and Conv individuals (Fig. 1b and Supplementary Fig. 2a). Importantly, the proportion of atMBC was significantly higher in patients who died than in those who survived (Fig. 1b). Conversely, the proportions and numbers of cMBC were significantly reduced in patients with severe COVID-19 compared with HD and Conv individuals. There was no significant difference between survivors and deceased patients at admission (Fig. 1c and Supplementary Fig. 2b). Changes in atMB and cMB cells were transient in subjects recovering from severe COVID-19. Thus, the frequency of atMBC significantly decreased and that of cMBC significantly increased during follow-up after recovery (Fig. 1d, e), suggesting that B-cell subset skewing was not pervasive in this clinical setting.

**Fig. 1:**
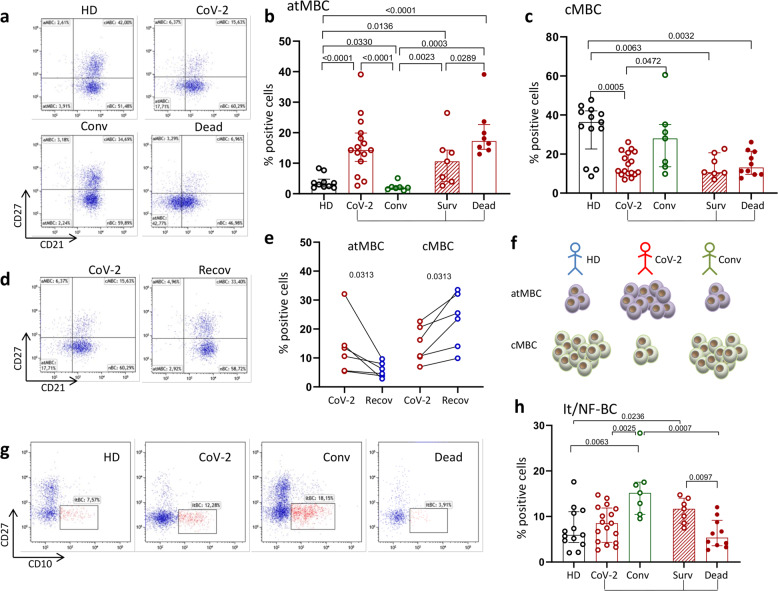
Changes in B-cell subpopulations during COVID-19. **a** Representative dot plots show the B-cell subset distribution in healthy donors (HD), all SARS-CoV-2-infected patients (CoV-2), convalescent subjects (Conv), and deceased (Dead) patients. **b**, **c** Frequencies of atypical memory B cells (atMBC) and classical memory B cells (cMBC), respectively, in HD, CoV-2, Conv, survivors (Surv) and dead patients. Middle bars represent medians with interquartile ranges. The Mann–Whitney *U* test was used to compare groups. **d** Representative dot plots showing B-cell subsets in the acute phase of COVID-19 (CoV-2) and after recovery (Recov). **e** Frequencies of atMBC and cMBC in patients in the acute phase of COVID-19 (CoV-2) and after recovery (Recov). Paired data were analyzed by the Wilcoxon signed rank test. **f** Cartoon summarizing the typical but transient skewed distribution of atMBC and cMBC during COVID-19. **g** Representative dot plots showing changes in immature transitional/newly formed B cells (it/NF-BC), identified as CD27-CD10+, in HD and patients. **h** Frequencies of it/NF-BC in HD and patients. Middle bars represent medians with interquartile ranges. The Mann–Whitney *U* test was used to compare the groups

Classical memory B cells are characterized by their ability to persist long term and rapidly respond upon rechallenge with the same pathogen.^6^ Most memory B cells in healthy humans express the cMBC phenotype (CD21^+^/CD27^+^). However, an atypical B-cell subset (atMBC) that downregulates or does not express these markers aberrantly appears during chronic infections including HIV,^7^ malaria,^8^ HCV,^3^ and HBV.^4^ The significance of atMBC arising during viral infections and whether this may be associated with impaired B-cell function are still controversial, and it is unclear whether these cells are also functionally defective in COVID-19. In line with this uncertainty, the expression levels of markers that have been associated with functional deficiency may vary greatly in atMBC from different individuals, supporting the hypothesis that atMBC is a heterogeneous population, probably influenced by the time since antigen exposure.^9^ Indeed, atMBC may be recently activated cMBC,^9^ which would make them refractory to further activation by antigen or other stimuli. Functional studies addressing whether B-cell stimulation with different SARS-CoV-2 proteins directly causes upregulation of inhibitory pathways in B cells, resulting in accumulation of atMBC that may be unable to neutralize the virus, are currently underway in our laboratory.

In addition to changes in atMBC frequencies, there were also differences in the proportion of immature transitional B cells (itBC) in the different groups. Even though no statistically significant differences were found between CoV-2 and HD, if we stratified the former according to recovery or death, deceased patients had significantly lower frequencies and numbers of itBC than survivors and Convs (Fig. 1g and Supplementary Fig. 2c). Intriguingly, survivors and Convs had significantly higher frequencies of itBC than HD. The developmental fate and role definition of itBC in humans is starting to unravel as they are now referred to as newly formed B cells (NF-BC), which, after leaving the bone marrow, evolve in the periphery into fully mature B cells.^10^ The role of these cells is still controversial, but growing evidence supports a role for it/NF-BC in protection against infections via the recognition of pathogen-associated molecular patterns and pathogen-associated antigens by differentiation into plasmablasts or IgG-secreting plasma cells that produce neutralizing antibodies.^10^ Hence, it is of interest that low frequencies of these cells in the peripheral blood are associated with poor outcomes, whereas higher frequencies characterize survivors and Conv donors of high-titer neutralizing antibody-containing plasma. It may thus be surmised that insufficient numbers of it/NF-BC are unable to produce enough neutralizing antibodies to control SARS-CoV-2 infection.

In conclusion, in this study, we provide novel insights into important changes in B-cell subpopulations during COVID-19 that may help to understand B-cell subset dynamics and to address more specific issues regarding their clinical significance and implications for therapeutic interventions.

## Supplementary information

Laboratory findings in patients with COVID-19 stratified according to clinical outcome

Supplementary Methods

Supplementary figure 1: Gating strategy to identify B cell subsets

Supplementary Figure 2: absolute numbers of B cell subsets per μL
