# Supplementary material for: Expansion of atypical memory B cells is a prominent feature of COVID-19
Source: Cell Mol Immunol. 2020 Sep 2;17(10):1101–3. doi: 10.1038/s41423-020-00542-2 (PMC7463104; doi:10.1038/s41423-020-00542-2)
Supplement: Supplementary file 1 — Laboratory findings in patients with COVID-19 stratified according to clinical outcome [file 41423_2020_542_MOESM1_ESM.docx]

**Supplementary Table 1.** Laboratory findings in patients with COVID-19 stratified according to clinical outcome.

|  |  |  | Total CoV-2 | Survived |  | Deceased | Convalescents |
| --- | --- | --- | --- | --- | --- | --- | --- |
|  | **Normal Range** |  | n=17  Median [range] | n=7  Median [range] |  | n=10  Median [range] | n=7  Median [range] |
| Males (n) |  |  | 13 | 5 |  | 8 | 7 |
| Females (n) |  |  | 4 | 2 |  | 2 | 0 |
| Age |  |  | 69 [32-92] | 55 [47-67] |  | 77 [53-92] | 81[56-92] |
| ALT U/ml | [11-34] |  | 29[11-140] | 42 [15-86] |  | 28 [13-68] | n.a. |
| AST U/ml | [11-39] |  | 39 [17-137] | 38 [17-51] |  | 51 [35-137] | n.a. |
| PLT x10^3^/µl | [150-450] |  | 219.5 [94-633] | 218 [98-633] |  | 193 [125-300] | 240 [196-299] |
| Lymphocytes c/µl° | [1500-4000] |  | 753 [446-1415] | 949 [731-1229] |  | 533 [446-1415] | 2400 [1500-3200] |
| LDH mU/ml | [125-220] |  | 359 [176-867] | 291 [176-410] |  | 491 [292-867] | n.a. |
| CRP mg/dl | <0.5 |  | 14.55 [0.01-36.36] | 8.84 [0.01-23.79] |  | 15.01 [2-36.36] | n.a. |
| INR. (%) | [0.9-1.2] |  | 1.11 [0.92-1.39] | 1.05 [0.92-1.2] |  | 1.13 [1-1.39] | n.a. |
| Creatinine mg/dl | [0.55-1.02] |  | 0.87 [0.51-2.91] | 0.75 [0.51-0.87] |  | 1.2 [0.73-2.91] | n.a. |

c/µl=cells/µl; ALT: Alanine Aminotransferase; AST: Aspartate Aminotransferase; PLT: Platelets; LDH: Lactate Dehydrogenase; CRP: C-reactive Protein; INR. International normalized ratio; n.a.: not available
