# Supplementary material for: Expansion of atypical memory B cells is a prominent feature of COVID-19
Source: Cell Mol Immunol. 2020 Sep 2;17(10):1101–3. doi: 10.1038/s41423-020-00542-2 (PMC7463104; doi:10.1038/s41423-020-00542-2)
Supplement: Supplementary file 2 — Supplementary Methods [file 41423_2020_542_MOESM2_ESM.docx]

Supplmentary Methods.

**Phenotypic analysis**

Peripheral blood monuclear cells (PBMC) were isolated by standard gradient centrifugation (Lympholyte^®^ - H, Cedarlane, Burlington, Canada) and stored in liquid nitrogen. Cryopreserved PBMC from patients and controls were thawed, washed with DPBS (CORNING, Manassas, VA, USA) supplemented with 2% fetal bovine serum (FBS; HyClone, GE Healthcare, South Logan, Utah, USA) and stained for phenotypic analysis using the following fluorochrome conjugated antibodies: CD27 BB515, CD19 BV605, CD10 BV421 and CD21 APC (all from BD Biosciences, San Diego, CA, USA). After 30’ of labeling, PBMC were washed, fixed in CellFix solution (BD Biosciences) and analyzed by FACS Celesta (BD Biosciences).

**Statistical Analysis**

To perform statistical analysis, GraphPad Software 8.01 (GraphPad Software Inc, La Jolla, CA). was used. Statistical differences between groups were assessed by the non-parametric Mann-Whitney U test. Paired data were analyzed by Wilcoxon signed rank test.
