# Supplementary figures and images for: Expansion of atypical memory B cells is a prominent feature of COVID-19
Source: Cell Mol Immunol. 2020 Sep 2;17(10):1101–3. doi: 10.1038/s41423-020-00542-2 (PMC7463104; doi:10.1038/s41423-020-00542-2)

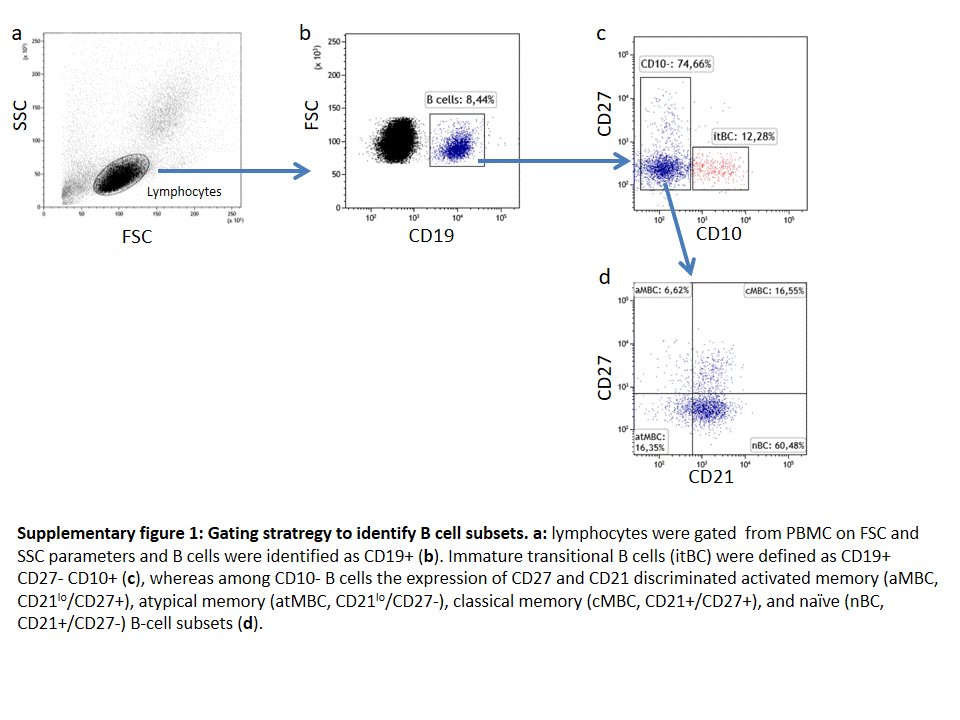

Supplement: Supplementary file 3 — Supplementary figure 1: Gating strategy to identify B cell subsets [file 41423_2020_542_MOESM3_ESM.gif]

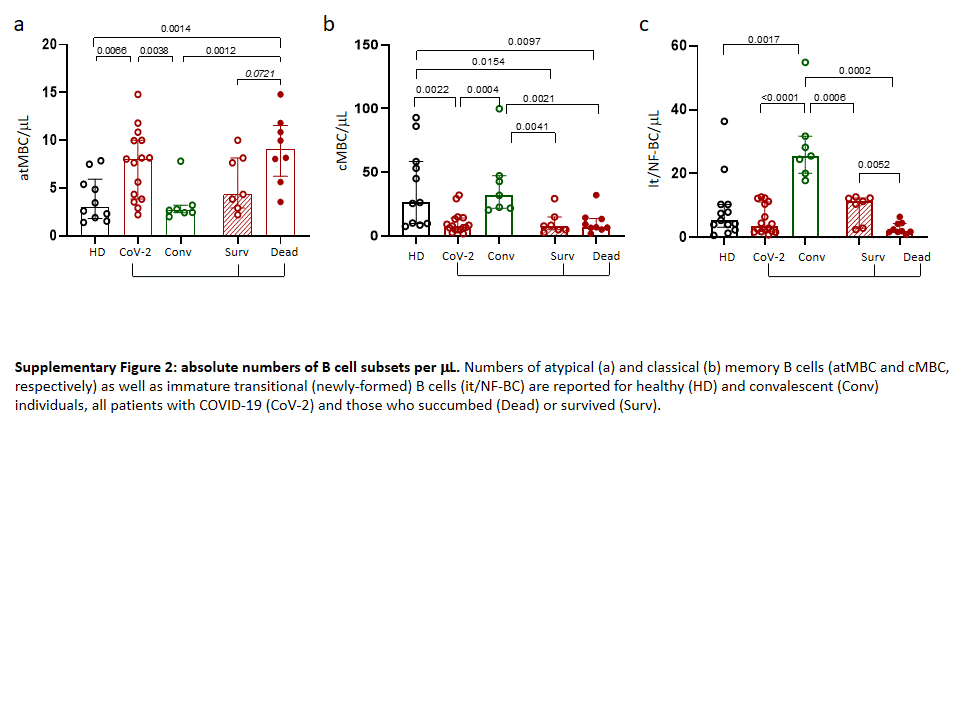

Supplement: Supplementary file 4 — Supplementary Figure 2: absolute numbers of B cell subsets per μL [file 41423_2020_542_MOESM4_ESM.gif]
